# Detection of DENV-4 genotype I from mosquitoes collected in the city of Manaus, Brazil

**DOI:** 10.1186/1743-422X-10-60

**Published:** 2013-02-19

**Authors:** Mario Luis Garcia de Figueiredo, Helda L Alfonso, Alberto Anastacio Amarilla, Luiz Tadeu Moraes Figueiredo, Victor Hugo Aquino, Cristóvão Alves da Costa, Sergio Luiz Bessa Luz

**Affiliations:** 1 Fundação Osvaldo Cruz – FIOCRUZ, Av. Teresina, 476 - Adrianópolis, CEP:(69.057-070, Manaus, AM, Brazil; 2 Department of Clinical, Toxicological and Bromatological Analysis, FCFRP/USP, Ribeirão Preto, SP, Brazil; 3 Virology Research Center, FMRP/USP, Ribeirão Preto, SP, Brazil; 4 National Institute for Research in the Amazon Region Manaus, Amazonas, Brazil

**Keywords:** Emerging flavivirus, Dengue serotype 4 in Brazil, RT-Hemi-Nested-PCR for Flavivirus

## Abstract

**Background:**

Dengue epidemics have been reported in Brazil since 1981. In Manaus, a large city in the Amazon region, dengue is endemic with all four-virus serotypes (DENV-1, -2, -3, and -4) simultaneously causing human disease. In 2008, during a surveillance of dengue virus in mosquitoes in the district of Tancredo Neves in Manaus, 260 mosquitoes of *Aedes* genus were captured, identified and grouped into pools of 10 mosquitoes.

**Findings:**

RNA extracts of mosquito pools were tested by a RT-Hemi-Nested-PCR for detection of flaviviruses. One amplicon of 222 bp, compatible with dengue virus serotype 4, was obtained from a pool of *Aedes aegypti*. The nucleotide sequence of the amplicon indicated that the mosquitoes were infected with DENV-4 of genotype I. This virus of Asian origin has been described in Manaus in 2008 infecting acute febrile illness patients.

**Conclusion:**

This is the first report of dengue virus serotype 4 genotype I infecting *Aedes aegypti* in the Americas.

## Findings

Dengue virus serotypes 1 through 4 (DENV-1, 2, 3 and 4) are the most important arboviruses worldwide based on number of cases and mortality. DENV infects approximately 50 million of people per year in 100 countries [1]. In Brazil, the incidence of dengue has been on an upward trend, and in the last decade 700,000 cases have been reported per year. Most Brazilian states are infested by *Aedes aegypti* and endure dengue transmission. The average age of dengue hemorrhagic fever patients has decreased in the last years, affecting a rising proportion of children. In recent years dengue outbreaks have included many atypical cases, including myocarditis, hepatitis, meningoencephalitis and acute kidney failure, and fatality rates have also increased [2,3].

DENV is transmitted to humans mainly by *Aedes aegypti* mosquitoes, which acquire the infection throught blood feeding on infected individuals or by transovarial transmission [4]. The first isolation of a DENV (DENV-1 and -4) in Brazil occurred in 1981, in the northern state of Roraima, from acute febrile patients [5]. Then, DENV-4 had disappeared from the country until 2008, when it was found infecting acute febrile patients in the city of Manaus, the capital of Amazonas state, which is located in the middle of the rain forest [6]. A phylogenetic analysis showed that the DENV-4 isolated in Manaus belonged to the genotype I, which has never been described in the American Continent [7]. In 2010, another DENV-4 genotype was detected in Roraima State; the genotype II, which has been circulating in Central America, the northern of South America and the Caribbean [8]. This DENV-4 genotype II rapidly spread through Brazil, producing outbreaks in the most populated areas of the northeastern and southeastern of the country (Ministry of Health of Brazil, 2012). Recently, the origin and evolution of DENV-4 genotypes (I and II) from northern and northeastern Brazil were analyzed [9]. We report here the first detection of DENV-4 genotype I infecting *Aedes aegypti* in the city of Manaus.

In 2008, as a part of a vector surveillance program for dengue virus detection, 203 *Aedes aegypti* and 57 *Aedes albopictus* were captured using adult mosquito traps in Tancredo Neves, a poor district of Manaus city. The mosquitoes were identified based on morphological characteristics in CO_2_ atmosphere and those from the same specie or genus, captured in the same place, were pooled (~10 adults/pool) based on day of collection. A volume of 1.5 ml PBS (pH7.8) containing 4% bovine albumin was added to each pool of mosquito. The insects were crushed using plastic pistils, and centrifuged at 2500x g for 30 minutes, at 4°C, to pellet the carcasses. The supernatant was split in 2 aliquots and stored at -80°C until use.

**Figure 1:**
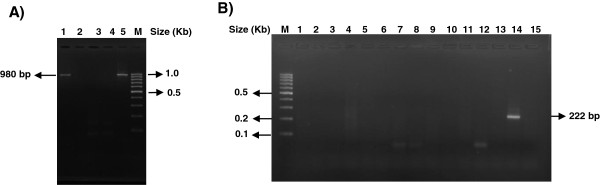
**Agarose gel electrophoresis 1.8% showing amplicons obtained by Flavivirus RT-PCR and Hemi-Nested-PCR from mosquitoes samples. A**) RT-PCR products from Aedes aegypti pools (lanes 1 to 3) plus negative and positive reactions control in lane 4 and 5, respectively. **B**) Hemi-Nested-RT-PCR products from pools of *Aedes aegypti *are in lanes 3 to 15 and negative reaction control in lane 1. M: molecular weight marker (100 bp).

The RNA from macerates of all mosquito pools was extracted using the Qiamp Viral RNA Mini Kit (QIAGEN, Germany). A RT-Hemi-Nested-PCR for detection of flaviviruses was used for testing these RNA extracts as previously reported by Bronzoni et al, 2005 [10]. This test allows virus preliminary identification based on amplicon size: 472 base pair (bp) for DENV-1, 316 bp for DENV-2, 628 bp for DENV-3, 222 bp for DENV-4, 253 bp for YFV and 232 bp for SLEV [10]. All molecular biology procedures were performed in order to avoid any type of contamination; different rooms were used for RNA purification, flavivirus genome amplification and PCR product analysis. A number of 21 pools of *Aedes aegypti* and 6 pools of *Aedes albopictus* were tested. One amplicon of 222 bp, compatible with DENV-4, was obtained from a pool of *Aedes aegypti* (Figure 1). The nucleotide sequence of 172 bp, of the amplicon obtained from RT-PCR, confirmed it was DENV-4. This amplicon was recovered from the agarose gel with the QUIAquick gel extraction kit (Qiagen, USA) and subjected to nucleotide sequencing with the ABI PRISM®3500 Genetic Analyzer (Applied Biosystems, Foster City, CA-USA). This sequence was named BR/AM/5B/2008 and registered in the European Nucleotide Archive (EMBL Nucleotide Sequence Database) with the accession number HE994136. The BR/AM/5B/2008 sequence was aligned with other 40 sequences belonging to DENV-4 isolated worldwide using the CLUSTAL W software [11]. This alignment was used for phylogenetic inference using the Neighbor-Joining method included in the MEGA 5 software [12]. The phylogenetic tree showed that the virus detected in this study belong to DENV-4 genotype I.

Vector surveillance is at the root of dengue control and prevention, with many methods available for detecting the presence of the mosquito vectors, *Aedes aegypti* and *Ae. albopictus*, in or around human residences. We show here an important result from a vector surveillance work performed in the city of Manaus where dengue is hyperendemic and has an almost continuous circulation of all 4 dengue virus serotypes [13].

**Figure 2:**
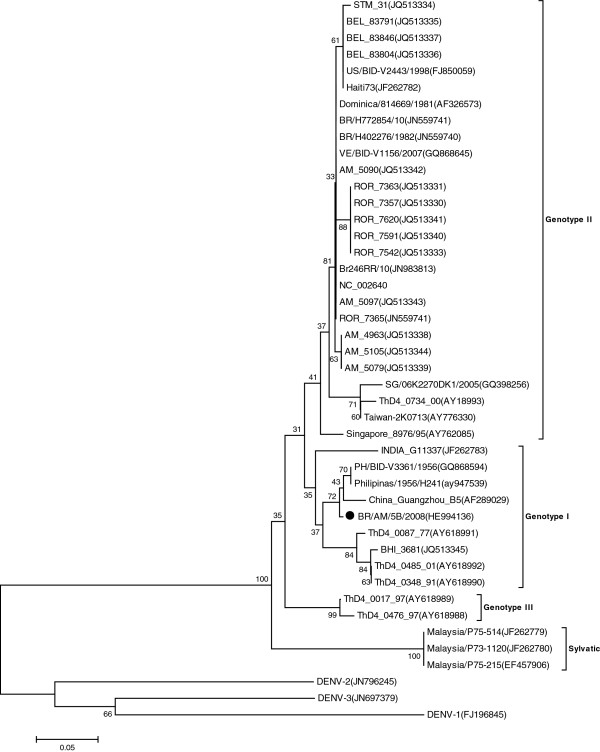
**DENV-4 phylogenetic tree based on the NS5 partial gene sequences. **The three was constructed using the Neighbor-joining method with 1000 bootstrap replications. DENV-1 to 3 was used as outgroup. Branch lengths are proportional to percentage of divergence. Tajima-Nei nucleotide substitution model was used with a gamma distribution (shape parameter = 1). The GenBank accession numbers, species, the country of origin, and year of isolation are shown.

DENV-4 was firstly described in Manaus in 2008 infecting patients with acute febrile illness [6]. Curiously it was found that this DENV-4 belonged to the genotype I, which is of Asian origin and was never described in the American Continent [6,7]. Phylogenetic analysis of core-prM junction of DENV-4 genotype I isolates from Manaus city showed that these isolates are grouped together with Philippines/1956/H241 (AY947539) and China_Guangzhou_B5 (AF289029) strains [7]. The report of DENV-4 genotype I in Manaus was seen with skepticism by the Brazilian Ministry of Health. However, probably, this virus might have been present in the region for some time, infecting patients and producing acute febrile illness but not spreading to other parts of Brazil [6]. The infection of *Aedes aegypti* captured in 2008 by DENV-4 of genotype I reported here support previous finding that this new virus was really presented in Manaus. The topology of the phylogenetic tree suggests the existence of two clades within genotype I, represented by Philippines and Thailand strains, respectively. The phylogenetic tree shows that the virus from Manaus reported here is closely related to those found in China and Philipines (Figure 2). Recently, 16 full-length sequences of DENV-4 from Brazilian isolates were obtained [9]. We include the new sequences of DENV-4 from Brazilian isolates available in Gene Bank. In our phylogenetic analysis we observed that BHI_3681(JQ513345) strain, isolated from human (autochthonous febrile patient in 2011 in Salvador, Bahia State) is phylogenetically related with our mosquitoes sample.

Interestingly, the topology of phylogenetic tree showed that BHI_3681(JQ513345) strain is most closely related to Thailand isolates. The introduction of DENV-4 genotype I might be related to trading among oriental companies that have facilities in the city of Manaus and receive company worker visits and imported containers from Asia. DENV-4 of genotype II has been also introduced in 2010 and presently, genotypes (I and II) of the virus are circulating in Manaus. This work also showed that the RT-PCR and Hemi-nested-PCR allowed the diagnosis of dengue virus infecting mosquitoes [4,10]. Therefore, it is a helpful tool for surveillance of dengue and other flaviviruses in vectors.

## Competing interests

The authors declare that they have no competing interests.

## Authors’ contributions

MLGF, HLA, AAA, LTMF, VHA, CAC and SLBL conceived of the study, and participated in its design and coordination. All authors read and approved the final manuscript.
